# Nano-Mechanical Properties and Creep Behavior of Ti6Al4V Fabricated by Powder Bed Fusion Electron Beam Additive Manufacturing

**DOI:** 10.3390/ma14113004

**Published:** 2021-06-01

**Authors:** Hanlin Peng, Weiping Fang, Chunlin Dong, Yaoyong Yi, Xing Wei, Bingbing Luo, Siming Huang

**Affiliations:** 1 Guangdong Provincial Key Laboratory of Advanced Welding Technologies, China-Ukraine Institute of Welding, Guangdong Academy of Sciences, Guangzhou 510650, China; henryhlpeng@163.com (H.P.); dongchl@gwi.dg.cn (C.D.); yiyy@gwi.dg.cn (Y.Y.); lbingbing11@126.com (B.L.); 2 School of Materials Science and Engineering, Huazhong University of Science and Technology, Wuhan 430074, China; weixing_hust@163.com; 3 School of Mechanical and Automotive Engineering, South China University of Technology, Guangzhou 510640, China; scutsiminghuang@163.com

**Keywords:** titanium alloys, electron beam additive manufacturing, nanoindentation, strain rate sensitivity, creep

## Abstract

Effects of scanning strategy during powder bed fusion electron beam additive manufacturing (PBF-EB AM) on microstructure, nano-mechanical properties, and creep behavior of Ti6Al4V alloys were compared. Results show that PBF-EB AM Ti6Al4V alloy with linear scanning without rotation strategy was composed of 96.9% α-Ti and 2.7% β-Ti, and has a nanoindentation range of 4.11–6.31 GPa with the strain rate ranging from 0.001 to 1 s^−1^, and possesses a strain-rate sensitivity exponent of 0.053 ± 0.014. While PBF-EB AM Ti6Al4V alloy with linear and 90° rotate scanning strategy was composed of 98.1% α-Ti and 1.9% β-Ti and has a nanoindentation range of 3.98–5.52 GPa with the strain rate ranging from 0.001 to 1 s^−1^, and possesses a strain-rate sensitivity exponent of 0.047 ± 0.009. The nanohardness increased with increasing strain rate, and creep displacement increased with the increasing maximum holding loads. The creep behavior was mainly dominated by dislocation motion during deformation induced by the indenter. The PBF-EB AM Ti6Al4V alloy with only the linear scanning strategy has a higher nanohardness and better creep resistance properties than the alloy with linear scanning and 90° rotation strategy. These results could contribute to understanding the creep behavior of Ti6Al4V alloy and are significant for PBF-EB AM of Ti6Al4V and other alloys.

## 1. Introduction

In recent years, additive manufacturing (AM) has attracted extensive attention and has great potential to be used in the fields of aerospace, automotive, energy, and medicine [1,2,3]. Powder bed fusion with electron beam (PBF-EB) AM is one of the AM methods, using a high-energy electron beam as a heat source to melt metal powders and then obtaining full-dense metallic components [4]. Compared with the selective laser melting (SLM) AM method, PBF-EB AM has several advantages [5]: (1) High vacuum condition is favorable for materials containing active elements; (2) high energy input is favorable for refractory materials. It has been reported that PBF-EB AM has been applied in fabricating high-entropy alloys [6], titanium alloys [7], and ceramics [8].

Due to their superior mechanical properties, excellent corrosion resistance, and outstanding biocompatibility, Ti6Al4V alloys have been used as biomedical implants and aviation materials [9,10,11,12]. However, the inherent difficulties in plastic processing and specific components with complex geometry make Ti6Al4V an attractive material for AM. The abrasion, fatigue, or impact properties of Ti6Al4V alloys could be further improved through AM [13,14]. In comparison with other AM methods, PBF-EB AM is the most widely-used process for producing Ti-based components because of their extremely high sensitivity to oxidation at high fabrication temperatures [15,16,17,18]. For AM Ti6Al4V alloys, the most attention is focused on the effects of PBF-EB AM parameters, post-treatment on microstructure, and mechanical properties. Tan et al. [19] investigated the columnar grain growth behavior of Ti6Al4V alloy during the PBF-EB AM process. Leon et al. [9] improved the mechanical properties of PBF-EB AM Ti6Al4V alloys using hot isostatic pressing post-treatment. Zheng et al. [20] investigated the effects of powder usage numbers on the hardness of PBF-EB AM Ti6Al4V alloys.

To date, only a few studies have been done to comprehensively assess the creep behavior of Ti6Al4V alloys [21]. However, the creep behavior is not completely understood. Investigation of creep behavior by traditional uniaxial tensile tests is time-consuming for lots of materials [22]. Nanoindentation tests are confirmed to be effective in analyzing the time-dependent plastic deformation of aluminum [23,24], high-entropy alloys [25,26], titanium alloys [27], and Ni_3_Al [28]. Although there is a bit of discrepancy between the indentation creep results and conventional uniaxial results [29,30,31], creep information including strain rate sensitivity, the creep stress exponent, and creep rate could be obtained based on the nanoindentation creep test [30,32]. Shen et al. claimed the discrepancy between nanoindentation and uniaxial methods results from the deformation mechanics [33]. The response from indentation creep tests thus includes the transient stage as well as the steady-state, or even post steady-state stages, which is more complex than that from uniaxial tests.

Wu et al. [34] found that the scanning strategy during selective laser melting AM has a great influence on residual stress of Ti6Al4V alloys. Tian et al. [35] reported that microstructure and mechanical properties of Ti6Al4V alloys could be influenced by the scanning strategy during selective laser melting AM. However, the reports on the effects of the scanning strategy during PBF-EB AM on nano-mechanical properties and creep behavior of Ti6Al4V alloys are rarely seen. Therefore, in this study, the effects of the scanning strategy during PBF-EB AM on microstructure, nanohardness, strain rate sensitivity, and creep behavior of Ti6Al4V are investigated.

## 2. Materials and Methods

### 2.1. Powder Preparation

**Figure 1:**
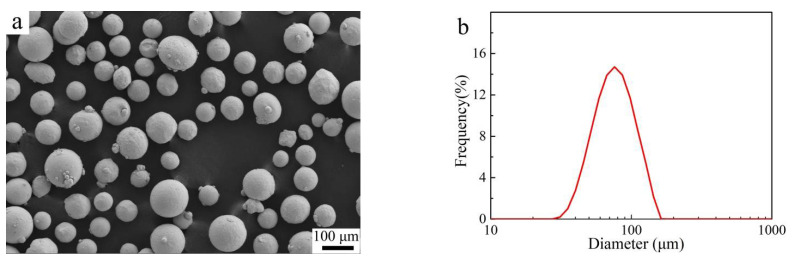
(**a**) SEM morphology and (**b**) particle size distribution of the as-received powders.

Spherical Ti6Al4V powders with an average size of 76 µm were provided by the supplier, Guangzhou Sailong Additive Manufacturing Co. LTD., Guangzhou, China, which was fabricated by plasma rotating electrode methods. Powder size distribution was characterized by a laser particle analyzer (MASTERSIZER 3000, Malvern, UK). A scanning electron microscope (SEM, ZEISS, Gemini SEM300, Oberkochen, Germany) equipped with energy-dispersive X-ray spectroscopy (EDS, Bruker, Billerica, MA, USA) was used to evaluate the chemical composition accuracy. The SEM morphology and particle size distribution of the as-received powders are shown in Figure 1a,b, respectively. The corresponding chemical composition was determined to be 5.85 ± 0.4 Al, 4.12 ± 0.24 V (wt.%), and balanced with Ti.

### 2.2. EB-PBF Processing

**Figure 2:**
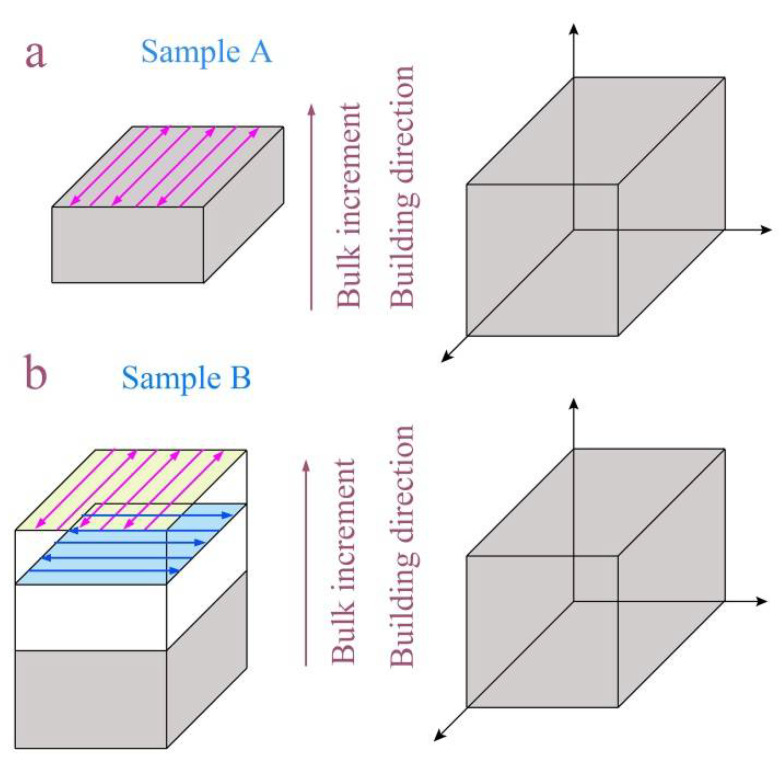
Schematic diagrams of building direction for PBF-EB AM Ti6Al4V alloys: (**a**) Sample A is horizontal back and forth linear scanning without rotation on the next layer; (**b**) Sample B is horizontal back and forth linear scanning with a 90° scan vector rotation on the next layer.

The cuboid-shaped sample with dimensions of 25 mm × 100 mm × 50 mm was produced using a T150 powder bed fusion electron beam additive manufacturing machine (Guangzhou Sailong Additive Manufacturing Co. LTD., Guangzhou, China). Plasma rotating electrode Ti6Al4V powders (mean diameter of 76 µm) were applied at a layer thickness of 50 µm. During the PBF-EB additive manufacturing, a scan speed of 5800 mm/s, a high voltage of 60 kV, a current of 14.5 mA, and a space of 100 µm between scan lines were adopted. The preheating temperature was kept at 730 °C for all of the samples to avoid powder smoke. Two scanning strategies were used in this study (details can be seen in Figure 2): (1) case (a) is horizontal back and forth linear scanning without rotation on the next layer, and the corresponding sample was referenced as sample A; (2) case (b) is horizontal back and forth linear scanning with a 90° scan vector rotation on the next layer, and the corresponding sample was referenced as sample B.

### 2.3. XRD Analysis

The block samples were cut along the building direction Z-axis using a wire electric discharge machine, 2 mm away from the base, and subsequently embedded for microstructural investigation and nanoindentation tests on the X-Y plane. The cross-sectioned samples were consecutively ground by #100, #600, #800, #1200, #1500, and #2000 grade silicon carbide papers to remove any surface oxides. Then ground samples were consecutively polished with 5 μm, 3 μm, 2 μm, 1 μm, and 0.5 μm grade diamond abrasive paste. Phase constituents were examined by X-ray diffraction (XRD, D/MAX-2500/PC; Rigaku Corp., Tokyo, Japan) with Mo Kα radiation. The angle range of 15–45° and a step size of 0.01° was adopted during the XRD test. More details can be seen in our previous research [36].

### 2.4. SEM Analysis

Before SEM observation, the polished block samples were etched with Kroll’s reagent (2% HF, 6% HNO3, and 92% H_2_O) for 5 s. The microstructure of the PBF-EB Ti6Al4V sample was observed by scanning electron microscope (SEM, FEI, QUANT250, Eindhoven, The Netherlands) equipped with energy-dispersive X-ray spectroscopy (EDS), with a working distance (WD) of 9.8 mm and high voltage of 10.00 kV. Additionally, the morphology and chemical composition of the raw powders were observed by scanning electron microscope (SEM, ZEISS, Gemini SEM300, Oberkochen, Germany) equipped with energy-dispersive X-ray spectroscopy (EDS, Bruker, Billerica, MA, USA). After the samples were electrolytically polished with 5% perchloric acid + 95% alcohol and electron backscattered diffraction (EBSD, FEI, Hillsboro, OR, USA) tests were performed to further investigate the microstructure. During the EBSD test, a step size of 0.1 μm was adopted for low magnification, and a step size of 0.01 μm was adopted for high magnification. During the EBSD test, a high voltage of 10.00 kV was used.

### 2.5. Nanoindentation Analysis

The nanomechanical properties and creep behavior of the PBF-EB AM Ti6Al4V alloys were characterized by nanoindentation tests (Hysitron, TI980, Minneapolis, MN, USA). All the tests were conducted at room temperature and performed with a three-sided Berkovich (CSM Instruments, Peuseux, Switzerland) diamond indenter. For investigation of the strain rate sensitivity, all nanoindentation tests were carried out at the same maximum load (10 mN) and with loading rates of 10, 1, 0.1, and 0.01 mN/s. The indenter was then held at the maximum load for 30 s, which was followed by unloading at a rate of 50 mN/s for all tests. For investigation of creep behavior, the specimen was loaded to different maximum loads, Pmax (10 mN, 20 mN, 50 mN, and 100 mN), at a successive loading rate of 0.5 mN/s and held at Pmax for 500 s.

## 3. Results

### 3.1. Microstructure

**Figure 3:**
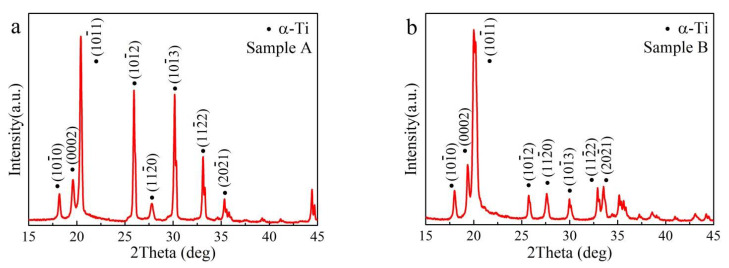
XRD pattern of PBF-EB AM Ti6Al4V alloy: (**a**) linear scanning strategy (sample A), (**b**) linear and 90° rotation scanning strategy (sample B).

XRD patterns of PBF-EB AM Ti6Al4V alloys (X-Y plane) arre shown in Figure 3, both for sample A with linear scanning strategy, and sample B with linear and 90° rotation scanning strategy. The XRD pattern confirms the dominant presence of an α-Ti phase, and this result is similar to the previous work [9]. Comparing the XRD results in Figure 3a,b, a tiny difference could be observed. For the XRD pattern of sample A (Figure 3a), the intensity of (10$\bar{1}$2), (10$\bar{1}$3), and (11$\bar{2}$2) diffraction peaks belonging to the α-Ti phase are stronger than those of sample B. This result indicates that the scanning strategy of PBF-EB AM probably leads to a difference in the texture of Ti6Al4V alloys.

**Figure 4:**
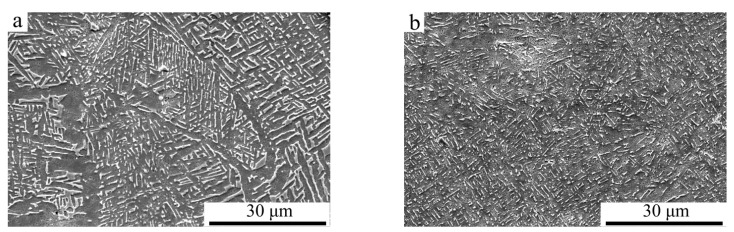
SEM microstructure of PBF-EB AM Ti6Al4V: (**a**) linear scanning strategy (sample A), (**b**) linear and 90° rotate scanning strategy (sample B).

As widely known, the microstructure of Ti6Al4V alloy consists of α-Ti and β-Ti phases [37]. During the PBF-EB AM process, the Ti6Al4V alloy originally solidifies into columnar β-Ti grains that grow along the build direction. As the additive manufactured alloys cool to a temperature below around 882 °C, the β-Ti grains boundaries act as nucleation sites for α-Ti grains, and the initial β-Ti within the grains transforms into α/β lamellar structures. This process promotes the development of β-Ti ribs surrounded by a continuous α-Ti phase [38]. The microstructures of sample A and sample B are shown in Figure 4a,b, respectively. It can be seen that the thickness of β-Ti ribs in sample B is smaller than those in sample A. Furthermore, β-Ti ribs in both sample A and sample B have a thickness of few than 1 μm.

The chemical composition of the a-Ti phase and β-Ti ribs in both sample A and sample B are listed in Table 1. The results are obtained by SEM/EDS. As widely known, Al is an α phase stabilizing element and usually diffused into α phase, while V is a β phase stabilizing element and usually diffused into the β phase [39,40]. It can be seen that there is no obvious enrichment or depth of Al and V elements in the α/β phase. For sample B, both the Al and V concentration in α phase and β ribs is very similar.

**Figure 5:**
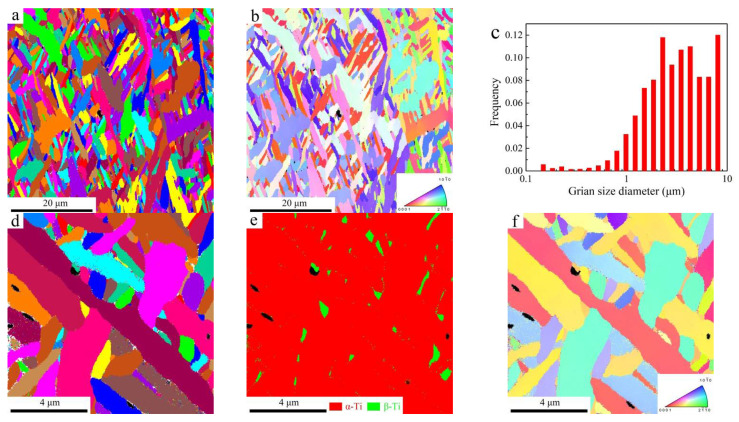
PBF-EB AM Ti6Al4V alloy with only the linear scanning strategy (sample A): (**a**) Grain size and (**b**) IPF images at low magnification; (**c**) grain size, (**d**) phase map, and (**e**) IPF figures at high magnification. The color of the inset figure in (**b**,**f**) represents grains orientation.

Since the conditions of heat accumulation and conduction are tightly linked to scanning strategies and relative position of the sample [4,34,41], the X-Y cross-section plane, 2 mm away from the base, was chosen for EBSD observation. Grain morphology and corresponding inverse pole figure (IPF) are shown in Figure 5a,b, respectively. The average grain size of the α-Ti phase is 2.3 µm, as shown in Figure 5c. To analyze the effects of scanning strategy on phase constitution of the PBF-EB AM Ti6Al4V alloy, a region with high magnification and a smaller step size (0.01 µm) was selected. The corresponding results are shown in Figure 5c,d. The phase maps are shown in Figure 5d. It can be seen that the PBF-EB AM Ti6Al4V alloy with only the linear scanning strategy (sample A) was composed of 96.9% α-Ti and 2.7% β-Ti.

**Figure 6:**
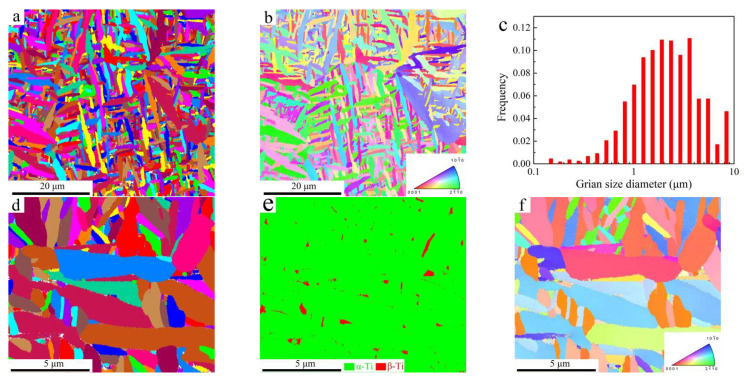
PBF-EB AM Ti6Al4V alloy with linear and 90° rotate scanning strategy (sample B): (**a**) Grain size and (**b**) IPF images at low magnification; (**c**) grain size, (**d**) phase map, and (**e**) IPF figures at high magnification. The color of the inset figure in (**b**,**f**) represents grains orientation.

For the PBF-EB AM Ti6Al4V alloy with linear and 90° rotation scanning strategy (sample B), the EBSD observation results are shown in Figure 6. From Figure 6c, it can be seen that the average thickness of the α-Ti phase is 2.5 µm. A high magnification was chosen with a step size of 0.01 µm. Additionally, the results are shown in Figure 6c,d. From the phase map (Figure 6e), it can be seen that the PBF-EB AM Ti6Al4V alloy with linear and 90° rotation scanning strategy (sample B) was composed of 98.1% α-Ti and 1.9% β-Ti. Compared with sample B, the α-Ti phase contents in sample A are a bit lower, while the β-Ti phase contents in sample A are a bit higher. The results indicate that scanning strategies during additive manufacturing could affect heat accumulation and conduction, and then influence the microstructure and mechanical properties.

From the EBSD analysis, the average size of the α-Ti phase in sample A is very similar to the α-Ti phase in sample A. Hence, the grain boundary strengthening is very similar due to the similar thickness of the α-Ti phase [42]. However, the solid solution strengthening is different. From Table 1, it can be seen that Al concentration in the α-Ti phase is similar in both samples. However, the V concentration in the α-Ti phase from sample A is higher than the value from sample B. Therefore, high V concentration in the α-Ti phase would lead to higher hardness.

### 3.2. Strain-Rate Sensitivity

Before the nanoindentation test, the Archimedes principle was employed to measure the porosity of PBF-EB AM Ti6Al4V alloys. The densities of sample A and sample B were 98.52% and 98.22%, respectively. It can be seen that the scanning strategy has little influence on the porosity of Ti6Al4V alloys under the selected additive manufacturing parameters.

**Figure 7:**
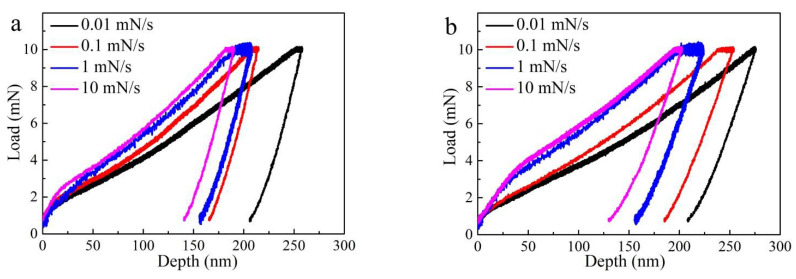
Typical load–depth curve with various strain rates at the same Pmax = 10 mN. (**a**) Linear scanning strategy (sample A), (**b**) linear and 90° rotation scanning strategy (sample A).

Figure 7 shows the typical load-depth curves for the PBF-EB AM Ti6Al4V alloy as obtained from nanoindentation tests under various loading rates. All nanoindentation tests were carried out at the same maximum load (10 mN). Loading rates of 0.01 mN/s, 0.1 mN/s, 1 mN/s, and 10 mN/s were applied, corresponding to strain rates of 0.001 s^−1^, 0.01 s^−1^, 0.1 s^−1^, and 1 s^−1^, respectively. As shown in Figure 7, the loading rates result in different depths. The depth increases significantly with the decreasing strain rate (or loading rates). Results from nanoindentation tests under different strain rates are presented in Table 2. It can be seen that as the strain rate increases in the range of 0.001–1 s^−1^, the hardness increases between 4.11 and 6.31 GPa for sample A. While for sample B, as the strain rate increases in the range of 0.001–1 s^−1^, the hardness increases between 3.98 and 5.52 GPa. The results indicate that the PBF-EB AM Ti6Al4V alloy with only the linear scanning strategy has higher nanohardness than the alloy with a 90° rotation scanning strategy.

The relationship between indentation nanohardness and strain rate could be expressed by Equation (1):

(1) $$H={C\;\dot{\varepsilon }}^{m}$$

where H is the nanohardness (GPa), *C* is the material constant, $\dot{\varepsilon }$ is the strain rate (s^−1^), and *m* is the strain rate sensitivity exponent [43]. Therefore, the strain-rate sensitivity exponent can be determined by the slope of an lnH vs. ln $\dot{\varepsilon }$ plot.

**Figure 8:**
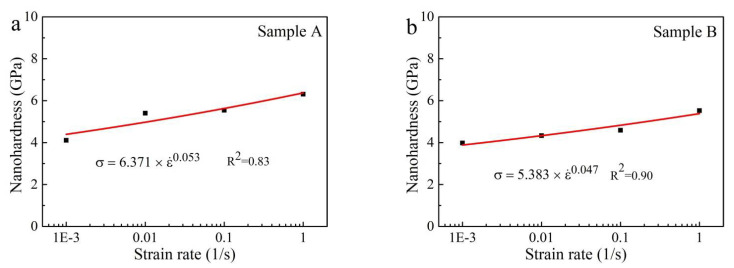
Representative nanohardness as a function of strain rate: (**a**) Linear scanning strategy (sample A) and the slope has a standard error of 0.327, (**b**) linear and 90° rotation scanning strategy (sample B) and the slope has a standard error of 0.181.

The relationship between indentation nanohardness (H) and strain rate ($\dot{\varepsilon }$) is shown in Figure 8. The equations in Figure 8a,b indicate that the relationship between H and $\dot{\varepsilon }$ has a high correlation coefficient, R^2^ = 0.83, and R^2^ = 0.9 for sample A and sample B, respectively. The results implied that this function can properly measure the dependence of the nanoindentation hardness on the strain rate. Based on the Figure 8, the strain-rate sensitivity exponent (*m*) are determined to be 0.053 ± 0.014 and 0.047 ± 0.009 for sample A and sample B, respectively.

The strain-rate sensitivity exponent (*m*) is important to evaluate the super-plasticity of materials. The strain-rate sensitivity exponent (*m*) of superplastic materials usually exceeds 0.3 [44]. The value of *m* (0.053 ± 0.014 and 0.047 ± 0.009) in this study does not exceed 0.1, which is similar to that of the most known materials. Yu et al. [43] determined the strain-rate sensitivity exponent of H13 tool steel to be 0.022 using nanoindentation. Jun et al. [27] characterized the strain-rate sensitivity exponent of Ti6246 alloys to be 0.005–0.039 via nanoindentation test.

### 3.3. Creep Behavior

**Figure 9:**
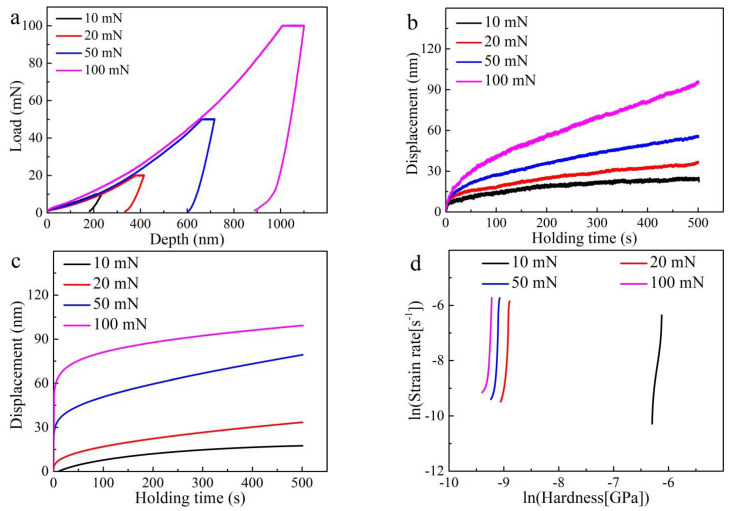
PBF-EB AM Ti6Al4V alloy with only the linear scanning strategy (sample A): (**a**) P–h curves under various maximum loads (10–100 mN); (**b**) experimental and (**c**) fitting creep displacement–time curves under various maximum loads (10–100 mN); (**d**) ln-ln plots of strain rate vs. nanoindentation stress under various maximum loads (10–100 mN).

For PBF-EB AM Ti6Al4V alloys with different scanning strategies, the typical load–displacement (P–h) curves under various maximum holding loads (Pmax) ranging from 10 to 100 mN are shown in Figure 9a. The curves consist of the loading stage, holding stage (creep stage), and unloading stage. It can be seen that plastic deformation occurred before the holding stage and the displacement increases during the holding stage. With the maximum holding load (Pmax) increased from 10 to 100 mN, the creep displacement dramatically increased. When the unloading is finished, the deformation is not completely recovered and indentation depth remains a large value. The nanoindentation results confirm the occurrence of creep behavior, despite the loading stress being very low at room temperature.

The creep behavior induced by the nanoindentation test has been explored by several scholars, and the creep displacement (*h*, nm) and holding time (*t*, s) could be depicted as follows [25,28,32]:

(2) $$h=h_{0}+a{\left(t-t_{0}\right)}^{b}+kt$$

where *h*_0_, *a*, *t*_0_, b, and *k* are constant and can be obtained by fitting the creep displacement (*h*) and holding time (*t*, s), *h*_0_ (nm), and *t*_0_ (s) refers to the displacement and time at the beginning stage of creep, respectively. After obtaining the relationship between creep displacement (*h*, nm) and holding time (*t*, s), the indentation strain rate ($\dot{\varepsilon }$, s^−1^) can be obtained according to the equation below [31]:

(3) $$\;\dot{\varepsilon }=\frac{1}{h}\frac{\mathrm{dh}}{\mathrm{dt}}$$

where displacement rate (dh/dt, nm/s) could be obtained by derivating the displacement–holding time (*h*–*t*). The nanoindentation stress (H, GPa) could be expressed as H = P/*h*^2^, in which P (mN) denotes the holding load and h (nm) refers to the indentation displacement. Then the creep stress exponent (*n*) could be calculated by the empirical equation below [25]:

(4) $$n=\frac{\partial \ln\;\dot{\varepsilon }}{\partial \mathrm{lnH}}$$

The experimental variations of displacement with time are shown in Figure 9b, corresponding to the holding stage in Figure 9a. Based on Equation (2), the fitting results of creep displacement (*h*)–time (*t*) curves are clearly shown in Figure 9c and a high correlation coefficient (R^2^ > 0.9) is obtained. From both experimental and fitting creep displacement-time results, it can be observed that the displacement increased dramatically with increasing loads. The creep displacement has a value of around 15 nm, 30 nm, 75 nm, and 95 nm, corresponding to the maximum holding load, Pmax, of 10 mN, 20 mN, 50 mN, and 100 mN, respectively. According to Equations (2)–(4), the creep stress exponent (*n*) under various indentation loads is shown in Figure 9d. The value of the creep stress exponent (*n*) decreased with the increasing maximum holding loads, Pmax, (10–100 mN).

**Figure 10:**
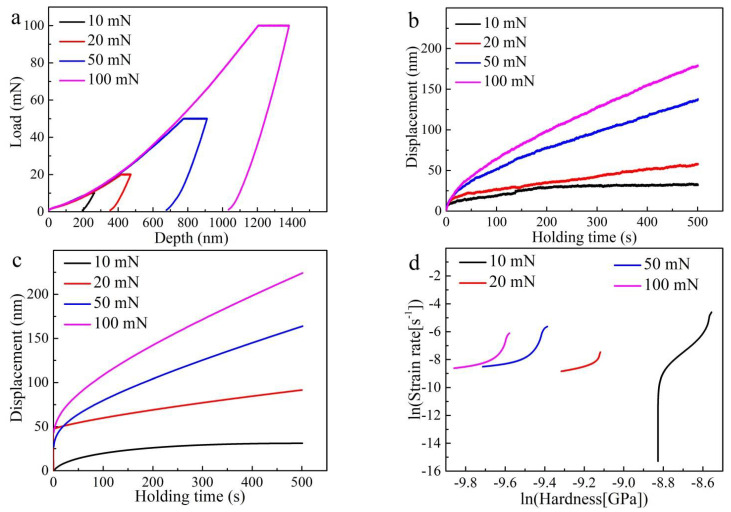
PBF-EB AM Ti6Al4V alloy with linear and 90° rotation scanning strategy (sample B): (**a**) P–h curves under various maximum loads (10–100 mN); (**b**) experimental and (**c**) fitting creep displacement–time curves under various maximum loads (10–100 mN); and (**d**) ln-ln plots of strain rate vs. nanoindentation stress under various maximum loads (10–100 mN).

For PBF-EB AM Ti6Al4V alloy with linear and 90° rotate scanning strategy (sample B), the typical load–displacement (P–h) curves under various peak loads ranging from 10 to 100 mN are shown in Figure 10a. The experimental and fitting creep displacement–time curves under various maximum loads (10–100 mN) are shown in Figure 10b,c, respectively. When fitting the creep displacement–time curves based on Equation (2), there is a high correlation coefficient (R^2^ > 0.9). When the maximum holding load (Pmax) increased from 10 to 20, 50, and 100 mN, the creep displacement had a values of around 25 nm, 85 nm, 170 nm, and 225 nm, respectively. Figure 10d shows the ln-ln plots of strain rate vs. nanoindentation stress under various maximum loads (10–100 mN). The value of the creep stress exponent (*n*) decreased with the increasing maximum holding loads, Pmax, (10–100 mN).

From both Figure 9b,c and Figure 10b,c, it can be seen that the creep curves from the nanoindentation test have transient-state creep and steady-state creep stages. As widely known, the classic creep curves obtained from uniaxial tensile tests have transient-state creep, steady-state creep, and accelerated creep stages [45,46]. This difference results from the very low loading stress during nanoindentation, which is unable to lead to the catastrophic failure of alloys. At the onset of nanoindentation creep behavior, it has a relatively high strain rate between 10^−3^ and 10^−4^ s^−1^, which belongs to the transient-sate creep stage. When the holding time is extended, the strain rate decreases to 10^−4^ s^−1^ and enters the steady-state creep stage.

The creep stress exponent (*n*) value is tightly linked to the creep mechanism. Usually, *n* = 1 indicates the diffusion creep mechanism, *n* = 2 indicates the grain boundary sliding mechanism, and *n* = 3–8 indicates the dislocation creep mechanism [23]. For the Ti6Al4V alloy (sample A), the creep stress exponent is calculated to be 39.491 ± 0.324 for 10 mN, 7.213 ± 0.017 for 20 mN, 4.201 ± 0.012 for 50 mN, and 2.556 ± 0.009 for 100 mN, respectively. Additionally, for the PBF-EB AM Ti6Al4V alloy (sample B), the creep stress exponent is calculated to be 11.89 ± 0.02 for 10 mN, 2.666 ± 0.004 for 20 mN, 1.572 ± 0.003 for 50 mN, and 1.842 ± 0.004 for 100 mN, respectively. The creep stress exponent of the PBF-EB AM Ti6Al4V alloy indicates that the creep behavior might be controlled by dislocation motion. The deformation induced by the indenter leads to a high density of dislocation and dislocation motion could effectively occur. Curiously, the calculated creep stress exponents decrease with increasing maximum loads (10–100 mN), which is opposite to the results of CoCrFeMnNi HEAs obtained by He et al. [23]. Lee et al. investigated nanoindentation creep behavior of CoCrFeMnNi high-entropy alloys and obtained creep stress exponent *n* = 14.34 for Pmax = 10 mN and *n* = 18.34 for Pmax = 50 mN [41].

## 4. Conclusions

In this study, the influences of scanning strategy during powder bed fusion electron beam additive manufacturing (PBF-EB AM) on microstructure, nano-mechanical properties, and creep behavior of Ti6Al4V alloy were compared. The results could contribute to understanding the creep behavior of Ti6Al4V alloy and are significant for PBF-EB AM of Ti6Al4V and other alloys. The conclusions can be summarized as follows:

Both PBF-EB AM Ti6Al4V alloys were composed of the predominant α-Ti phase and barely β-Ti phase. Alloys with only the linear scanning strategy were composed of 96.9% α-Ti and 2.7% β-Ti phases, while alloys with linear and 90° rotation scanning strategy were composed of 98.1% α-Ti and 1.9% β-Ti phases. Additionally, the thickness of β ribs in alloys with only the linear scanning strategy are a bit larger than those in the sample with the linear and 90° rotation scanning strategy, but both have a value of lower than 1 μm.

The nanohardness of the PBF-EB AM Ti6Al4V alloy with linear scanning strategy is a bit higher than the value for alloys with linear and 90° rotation scanning strategy. The nanoindentation hardness increased by a range of 4.11–6.31 GPa and 3.98–5.52 GPa with an improvement in the strain rate ranging from 0.001 to 1 s^−1^. The alloy with only the linear scanning strategy and 90° rotation scanning strategy has a strain-rate sensitivity exponent *m* = 0.053 ± 0.014 and *m* = 0.047 ± 0.009, respectively.

The PBF-EB AM Ti6Al4V alloy with only the linear scanning strategy has better creep resistance properties than the alloy with a 90° rotation scanning strategy. Increasing peak holding load (10–100 mN) led to the dramatic increment of creep displacement (15–95 nm and 25–225 nm) and the creep behavior was mainly dominated by dislocation motion during deformation induced by the indenter.

## Figures and Tables

**Table 1:** Chemical composition of α-Ti phase and β-Ti ribs in PBF-EB AM Ti6Al4V with different scanning strategies.

Chemical Composition	Sample A		Sample B	
	α-Ti (wt%)	β-Ti ribs (wt %)	α-Ti (wt %)	β-Ti ribs (wt %)
Al	5.48 ± 0.64	5.86 ± 0.50	5.48 ± 0.07	5.32 ± 0.65
V	4.05 ± 2.55	2.83 ± 0.54	3.22 ± 0.66	2.93 ± 0.66
Ti	90.48 ± 1.92	91.32 ± 0.04	91.2 ± 0.59	90.25 ± 2.14

**Table 2:** Various loading rates, strain rates, and corresponding nanohardnesses for PBF-EB AM Ti6Al4V alloys with different scanning strategies.

Loading Rate (mN/s)	Strain Rate (s^−1^)	Hardness (GPa)	
		Sample A	Sample B
0.01	0.001	4.11	3.98
0.1	0.01	5.4	4.33
1	0.1	5.54	4.59
10	1	6.31	5.52

## Data Availability

The data presented in this study are available on request from the corresponding author after obtaining permission of authorized person.
